# Prognostic impact of pre- and post-bronchodilator airflow obstruction and post-bronchodilator reference values in a population-based study. On behalf of the PLATINO group*

**DOI:** 10.36416/1806-3756/e20250275

**Published:** 2026-05-22

**Authors:** Rogelio Pérez-Padilla, Maria Montes de Oca, Ireri Thirion-Romero, Maria Victorina Lopez, Jose R. Jardim, Adriana Muino, Gonzalo Valdivia, Ana Maria B. Menezes

**Affiliations:** 1. Instituto Nacional de Enfermedades Respiratorias, Ciudad de México, México.; 2. División de Neumología, Hospital Universitario de Caracas, Universidad Central de Venezuela y Centro Médico de Caracas, Caracas, Venezuela.; 3. Universidad de la República, Hospital Maciel, Montevideo, Uruguay.; 4. Escola Paulista de Medicina, Universidade Federal de São Paulo, São Paulo (SP) Brasil.; 5. Facultad de Medicina, Pontificia Universidad Católica de Chile, Santiago, Chile.; 6. Programa de Pós-Graduação em Epidemiologia, Universidade Federal de Pelotas, Pelotas (RS) Brasil.

**Keywords:** Pulmonary disease, chronic obstructive, Reference values, Spirometry, Airflow obstruction, chronic, Mortality

## Abstract

**Introduction:**

Post-bronchodilator (BD) spirometry testing is required for a diagnosis of airflow obstruction (AO) and COPD. We compared the impact of pre- and post-BD AO, as well as that of pre-BD and post-BD reference values, on survival, exacerbations, and FEV_1_ decline.

**Methods:**

We analyzed data derived from the Proyecto Latinoamericano de Investigación en Obstrucción Pulmonar (PLATINO, Latin American Project for the Investigation of Obstructive Lung Disease) study, involving individuals residing in three Latin American cities and evaluated 5-9 years after baseline examination. Categories were formed by pre-and post-BD FEV_1_/FVC < the 5th percentile (lower limit of normal) by PLATINO reference values (pre-BD and post-BD).

**Results:**

At baseline, 2,942 participants completed pre- and post-BD spirometry; 2,262 were normal (controls); 139 had pre-BD AO (FEV_1_/FVC below the lower limit of normal; reversible AO); 230 had pre-BD and post-BD AO (persistent AO); 43 had only post-BD AO; and 148 had a preserved ratio impaired spirometry (PRISm) pattern. Additionally, 105 individuals had post-BD AO by post-BD reference values. When compared with controls, the reversible AO group (hazard ratio [HR] = 1.9; 95% CI, 1.1-3.1), the persistent AO group (HR = 2.99; 95% CI, 2.1-4.3), the PRISm group (HR = 1.6; 95% CI, 0.9-2.7), and the group of patients with AO by post-BD reference values (HR = 1.9; 95% CI, 1.1-3.4) had a higher mortality; those with a PRISm pattern and those with persistent AO had more exacerbations, and the latter group had an additional FEV_1_ decline in adjusted models (−13.4 mL/year; 95% CI, −6 to −21). The reversible AO group had a higher risk of developing COPD (post-BD AO) during follow-up (OR = 4.1; 95% CI, 2.0-8.5).

**Conclusions:**

Individuals with post-BD AO identified only with post-BD reference values had an increased risk of death. Those with pre-BD AO had higher mortality and an increased risk of developing COPD, therefore requiring close follow-up monitoring and being classified as pre-COPD patients.

## INTRODUCTION

Although it is accepted that the spirometric criterion for defining airflow obstruction (AO) and COPD is a low FEV_1_/FVC ratio after the use of a bronchodilator (BD), the abnormal threshold or cutoff point remains a topic of controversy. For several years, the GOLD criterion for confirming the diagnosis of COPD has been a post-BD FEV_1_/FVC ratio of < 0.70,^1^ ^,^ ^2^ whereas recommendations for the spirometry interpretation^3^ ^,^ ^4^ define AO as an FEV_1_/FVC ratio below the 5th percentile (lower limit of normal-LLN). There are a significant number of pre-BD spirometric equations with large variations in predicted values for individuals of the same age, sex, and height, which can probably generate interpretation errors. Although the Global Lung Function Initiative^5^ ^,^ ^6^ has generated compiled reference values to improve heterogeneity, these values are unlikely to properly fit populations with varied ethnic compositions, altitudes of residence, environmental exposures, and nutrition levels, among other factors that may affect lung function.^5^ ^,^ ^7^

Several spirometric measurements tend to increase after BD use including the 5th percentile (LLN), and reference values obtained from post-BD testing would represent a more appropriate LLN to maintain the arbitrary but commonly agreed 5% of false positives in a healthy population. The heterogeneity of reference values does not affect COPD diagnosis by the GOLD criteria; it only affects the determination of AO severity. However, with the LLN cutoff criterion, the variations in the reference values may also lead to misclassification in the diagnosis of COPD. Few post-BD reference values have been reported. This increases the difficulty in adequately interpreting spirometry.^8^ ^-^ ^11^ In a previous study, we reported that the prevalence of AO increased using the post-BD LNN criterion with the post-BD testing reference values compared to the result of a post-BD test compared to pre-BD reference values (10.8% vs. 7.6% with FEV_1_/FVC < LLN).^12^

More recently, the need for post-BD spirometry to diagnose COPD has been challenged^13^ ^,^ ^14^ by showing that individuals with pre-BD AO have adverse outcomes and a higher frequency of developing post-BD AO that in the presence of compatible symptoms or exposures define COPD as suggested by GOLD.^1^

The *Proyecto Latinoamericano de Investigación en Obstrucción Pulmonar* (PLATINO, Latin American Project for the Investigation of Obstructive Lung Disease) study evaluated the prevalence of individuals with FEV_1_/FVC and FEV_1_/FEV_6_ below the 5th percentile (LLN) using internally derived reference values for tests done before and after BD administration, showing that the prevalence of AO by the fixed ratio or the LLN decreased approximately one third on post-BD tests when compared with pre-BD tests^12^ and that the use of post-BD reference values doubled the prevalence of post-BD AO in the general population. 

The present study used longitudinal data from the PLATINO study, conducted in three Latin American cities (Montevideo, Uruguay; Santiago, Chile; and São Paulo, Brazil)^15^ to evaluate the implications of having pre-BD spirometric AO and the advantages of utilizing post-BD reference values. We analyzed the general characteristics, survival, exacerbation risk, and lung function decline of the subpopulations of patients with pre-BD AO and post-BD AO compared with internally defined pre- and post-BD reference values.^8^

## METHODS

### Study design and population

The detailed methods of the PLATINO baseline and follow-up studies have been described elsewhere.^15^ ^,^ ^16^ The PLATINO study was a population-based study conducted in five Latin American cities (Montevideo; Santiago; São Paulo; Mexico City; and Caracas, Venezuela) from 2002 to 2004 among adults ≥ 40 years of age.^16^ The study was conducted in two phases: 1) the baseline survey in the five centers^15^ ^,^ ^16^; and 2) the follow-up visit that occurred in three of the five centers (Montevideo, Santiago, and São Paulo) 5, 6, and 9 years after the baseline surveys, respectively.^15^

The baseline study sample (with post-BD spirometry testing) consisted of 5,183 individuals (Montevideo: 884; Santiago: 1,140; São Paulo: 918; Mexico City: 964; and Caracas: 1,277), and the prevalence of a post-BD FEV_1_/FVC ratio of < 0.70 ranged from 7.8% to 19.7%. At follow-up, individuals from the original baseline sample in Montevideo, Santiago, and São Paulo were located, were visited at their homes, were re-interviewed, and underwent post-BD spirometry. In addition, mortality data were prospectively collected from the time of the baseline visit to the follow-up visit. 

Ethical approval was obtained from the institutional review boards at all five sites (the *Instituto Nacional de Enfermedades Respiratorias*, in Mexico City; the *Universidad Central de Venezuela*, in Caracas, Venezuela; the *Pontificia Universidad Católica de Chile*, in Santiago, Chile; the *Universidade Federal de Pelotas*, in the city of Pelotas, Brazil; the *Universidade Federal de São Paulo*, in the city of São Paulo, Brazil; and the *Universidad de la República*, in Montevideo, Uruguay) for the baseline evaluation and the three sites participating in the follow-up study. All participants gave written informed consent. 

### Assessments and measurements

The PLATINO questionnaire is available at https://platino.alatorax.org. The questionnaire was used at the PLATINO baseline and follow-up visits. Spirometry was undertaken on individuals who did not meet any of the exclusion criteria (99% of the sample), with the use of an ultrasonic spirometer (EasyOne; ndd Medical Technologies, Zurich, Switzerland). Spirometry was performed before BD administration and 15 min after administration of 200 µg of albuterol, in accordance with the 2005 American Thoracic Society criteria of acceptability and reproducibility.^17^

Spirometric abnormalities were defined with pre-BD and post-BD measurements compared with the LLN of the PLATINO pre-BD reference values^18^ and post-BD reference values,^8^ as follows: 

normal spirometry-FEV_1_/FVC, FEV_1_, and FVC at or above the LLN for reference values 

AO (FEV_1_/FVC < LLN for pre-BD reference values) on pre-BD tests but not on post-BD tests (the reversible AO group) 

AO on pre-BD and post-BD tests, i.e., persistent AO with the use of the pre-BD reference values. This is the group fulfilling the current criteria for COPD, utilizing the LLN cutoff point and the most commonly used reference values obtained from pre-BD tests. 

AO on post-BD tests but not on pre-BD spirometry with the use of the pre-BD reference values. This group would be identified only if a post-BD test were done, even if the pre-BD test were normal. 

AO on post-BD tests only if compared with post-BD reference values. This group would be identified only by utilizing post-BD reference values. 

preserved ratio impaired spirometry (PRISm)-normal post-BD FEV_1_/FVC (non-obstructive), but FEV_1_ or FVC < LLN either on pre- or post-BD tests, found predictive of adverse outcomes^19^ and increased risk of developing COPD,^1^ ^,^ ^19^ and therefore required to be excluded from normal spirometries 

Exacerbation was self-reported and defined by symptoms (deterioration of breathing symptoms that affected usual daily activities or caused missed work) within the 12 months preceding the study; however, the severity of each exacerbation was not specified. 

### Statistical analysis

The primary comparison was between the reversible and persistent AO groups, and PRISm patterns, with those with normal spirometry as controls. ANOVA was used, with a Bonferroni adjustment for multiple comparison tests, for quantitative variables. The chi-square test was used for nominal variables as a general description of the groups but with particular interest in those related to well-known risk factors for COPD: tobacco smoking (pack-years and current smoking); a previous diagnosis of asthma; tuberculosis; occupational exposure or biomass exposure; level of education; having family members with COPD; hospitalization in childhood for respiratory problems; and number of exacerbations. 

Survival among the groups was compared by means of Cox proportional hazards models. Lung function decline was assessed by multiple regression models (only in individuals with spirometric tests in both evaluations). A self-report of two or more exacerbations in the previous year was assessed by logistic regression models. The results of the models were described crude and adjusted for covariates such as age, sex, current smoking (expressed as yes or no), cumulative smoking in pack-years, BMI > 30 kg/m^2^, number of years of schooling, and previous medical diagnosis of respiratory diseases or other comorbidities, as determined by a questionnaire. 

## RESULTS

### Baseline characteristics

Of the 3,151 study participants in Santiago, Montevideo, and São Paulo, 2,942 underwent post-BD spirometry at baseline (Table 1), 2,815 were evaluated at follow-up, and 2,136 also underwent post-BD spirometry at follow-up (858 participants in Santiago, 683 in Montevideo, and 595 in São Paulo-the population that was utilized for analysis of lung function decline). A total of 301 deaths were documented. Follow-up rates for each independent variable category were around 80%.^15^

**Table 1:** Baseline characteristics of the study population, by lung function category.^a^

Variables	Normal (n = 2,253)	Reversible AO (pre-BD reference values) (n = 139)	Persistent AO (pre-BD reference values) (n = 230)	Post-BD AO but no pre-BD AO (pre-BD reference values) (n = 43)	Post-BD AO (post-BD reference values) (n = 105)	Pre- and post-BD PRISm (n = 157)
% of males†	39 (37-41)	32 (25-41)	57 (50-63)	33 (20-48)	66 (56-74)	54 (46-61)
Age, years^‡^	57 ± 12	57 ± 12	62 ± 12	58 ± 11	57 ± 10	55 (11)
Height, cm^‡^	160 ± 10	159 ± 9	161 ± 9	157 ± 9	166 ± 10	163 (11)
BMI, kg/m^2^*^‡^	28 ± 5	28 ± 5	26 ± 5	28 ± 6	28 ± 5	29 (6)
% of current smokers^†^	29 (27-31)	37 (30-46)	47 (41-54)	42 (28-58)	47 (37-56)	34 (27-42)
% of exposure to occupations with dust (≥ 10 years)	31 (29-33)	29 (22-38)	44 (38-51)	30 (18-46)	40 (31-50)	39 (31-47)
% of individuals with ≥ 2 exacerbations in the previous year†	2 (1-3)	6 (3-12)	7 (4-11)	2 (0-16)	3 (1-9)	3 (1-7)
% of history of asthma^†^	13 (11-14)	27 (20-35)	33 (27-39)	26 (14-41)	18 (12-27)	17 (11-23)
% of history of COPD^†^	3 (2-4)	6 (3-12)	16 (12-21)	9 (3-23)	8 (4-15)	8 (4-13)
% of previous tuberculosis^†^	3 (2-3)	4 (2-9)	10 (6-14)	2 (0-16)	5 (2-11)	4 (2-9)
% of chronic bronchitis (cough or phlegm)^†^	10 (9-11)	12 (7-18)	29 (23-35)	14 (6-28)	13 (8-21)	17 (12-24)
% of use of any respiratory medication^†^	15 (13-16)	18 (12-25)	33 (28-40)	16 (8-31)	21 (14-30)	17 (11-23)
% of missed work in the previous year^†^	11 (10-12)	19 (13-26)	23 (18-30)	23 (13-39)	12 (7-20)	18 (13-25)
% of self-perception of good or excellent health^†^	73 (71-75)	65 (56-72)	64 (58-70)	67 (52-80)	71 (62-79)	62 (54-69)
% of obesity^†^	32 (30-34)	29 (22-37)	20 (15-25)	33 (20-48)	24 (17-33)	38 (31-46)

AO: airflow obstruction; BD: bronchodilator; and PRISm: preserved ratio impaired spirometry. ^a^Data presented as mean ± SD for continuous variables or as percent prevalence (95% CI). *p < 0.05 (ANOVA). ^†^p < 0.05 (chi-square test). ^‡^p < 0.05 (ANOVA Bonferroni-analysis of variance and covariance with Bonferroni multiple-comparison test only for age, height, BMI, and exacerbation in the previous year).

**Figure 1:**
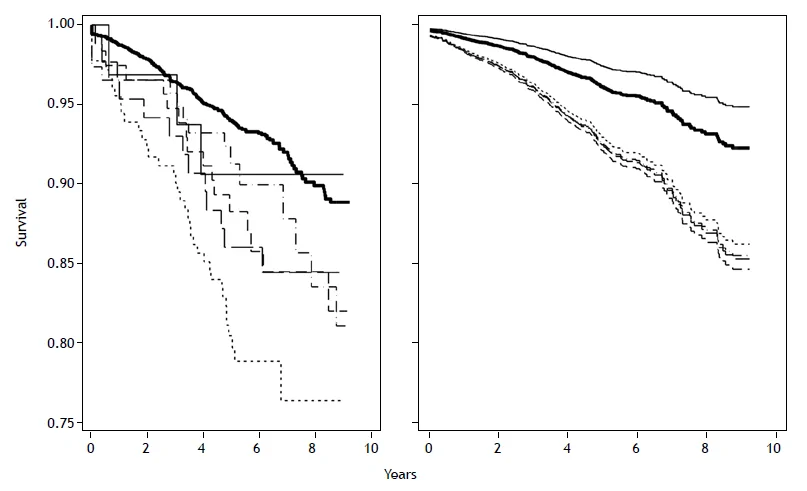
Survival curves for the study groups. In A, crude survival curves. In B, survival curves adjusted for age, sex, comorbidities, exacerbations, and smoking. The continuous thick line represents the control group (normal spirometry). The thin dashed line represents the reversible airflow obstruction (AO) group, showing decreased survival. The thin short-dash line represents the persistent AO group, being compared with pre-bronchodilator (BD) reference values. The long-and-short-dash line represents the post-BD AO group, being compared with post-BD reference values. The thin long-dash line represents the preserved ratio impaired spirometry group, overlapping in the adjusted model (in B). Change in survival vs. the control group was not statistically significant for the group of patients with post-BD AO but no pre-BD AO (thin, tight dot curve).

The baseline clinical and functional characteristics in the study groups are shown in Tables 1 and 2, respectively, as well as in Table S1 (supplementary material). Of the 2,942 participants with post-BD spirometry at baseline, 2,253 (77%) had normal spirometry,139 (4.8%) were in the reversible AO group, 230 (7.9%) were in the persistent AO group, 43 (1.5%) had post-BD AO but no pre-BD AO, 105 (3.6%) had post-BD AO only if compared with reference values derived from post-BD tests, and 157 (5.4%) were in the PRISm group (Table 1 and Figure 1). 

**Table 2:** Baseline lung function of the study population, by lung function category.^a^

Variables	Normal (n = 2,253)	Reversible AO (pre-BD reference values) (n = 139)	Persistent AO (pre-BD reference values) (n = 230)	Post-BD AO but no pre-BD AO (pre-BD reference values) (n = 43)	Post-BD AO (post-BD reference values) (n = 105)	Pre- and post-BD PRISm (n = 157)
Pre-BD FEV_1_, L	2.72 ± 0.75	2.19 ± 0.69	1.85 ± 0.74	2.24 ± 0.77	2.63 ± 0.74	1.99 ± 0.66
Post-BD FEV_1_, L	2.80 ± 0.76)	2.42 ± 0.72)	1.97 ± 0.74)	2.31 ± 0.71)	2.75 ± 0.74	2.15 ± 0.66
Pre-BD FVC, L	3.55 ± 0.97	3.45 ± 1.05	3.35 ± 1.10	3.16 ± 1.09	3.99 ± 1.11	2.60 ± 0.85
Post-BD FVC, L	3.50 ± 0.94	3.30 ± 0.97	3.47 ± 1.11	3.65 ± 1.07	4.06 ± 1.05	2.71 ± 0.82
Pre-BD FEV_1_/FVC, %	77 ± 6	63 ± 5	54 ± 9	71 ± 7	66 ± 6	77 ± 7
Post-BD FEV_1_/FVC, %	80 ± 5	73 ± 5	56 ± 9	63 ± 4	68 ± 3	79 ± 6
Good quality pre-BD test, %*	96 (95-97)	92 (86-96)	95 (92-97)	81 (66-91)	90 (83-95)	96 (92-98)
Good quality post-BD test, %*	96 (95-96)	93 (87-96)	95 (92-97)	84 (69-92)	93 (87-97)	97 (93-99)
Pre-BD FET, s	11.0 ± 3.3	15.0 ± 4.5	16.4 ± 5.1	11.6 ± 4.4	14.0 ± 4.5	8.9 ± 3.2
Post-BD FET, s	9.58 ± 2.7	11.2 ± 3.2	15.5 ± 4.6	15.3 ± 4.5	13.8 ± 3.4	8.3 ± 2.5
Flow responders**	6.1 (5.2-7.2)	31.7 (24-40)	17.4 (13-23)	13.9 (6.3-28)	12.3 (7.3-20)	19.1 (14-26)
Volume responders^†^	3.9 (3.1-4.7)	2.9 (1.1-7.5)	18.7 (14-24)	67.4 (52-80)	13.3 (8-21)	16.6 (11-23)

AO: airflow obstruction; BD: bronchodilator; PRISm: preserved ratio impaired spirometry; and FET: forced expiratory time. ^a^Data presented as mean ± SD for continuous variables or as percent prevalence (95% CI). *p < 0.05 (chi-square test). **Increase in FEV_1_ by 10% of the predicted value after BD administration. ^†^Increase in FVC by 10% of the predicted value after BD administration.

The patients with persistent AO were more likely to be older; were more likely to be male; were heavier smokers; more often reported occupations with dust exposure and two or more exacerbations in the previous year; and were more likely to report the following: a previous diagnosis of asthma; COPD; depression; having little energy; a lower perception of good health; and more use of respiratory medications. The PRISm, post-BD AO (when compared with post-BD reference values), and post-BD but no pre-BD AO groups had those variables in an intermediate position when compared with the normal group, the exception being obesity and diabetes, which were more common in the post-BD but no pre-BD AO and PRISm groups. In the former, the quality of spirometry was lower, with longer forced expiratory time on the post-BD test and a more common and intense increase in FVC after BD administration (volume responders). 

### Baseline lung function

At baseline, as expected, FEV_1_/FVC and FEV_1_ were lower in the persistent AO group and higher in the PRISm and normal groups, whereas FVC was lowest in the PRISm group. FEV_1_ after BD administration increased more in the reversible AO group (flow responders). The patients with AO on post-BD tests but not on pre-BD spirometry had a higher FVC increase after BD administration (volume responders), with a longer forced expiratory time and more heterogeneity in test quality. The proportion of good quality in post-BD spirometry was similar in all groups except for the group of patients with post-BD AO but no pre-BD AO, with longer forced expiratory time (Table 2). 

### Survival, exacerbations, and lung function decline

Table 3 describes the association of the different groups at baseline with survival, exacerbations in the previous year, and lung function decline in the follow-up evaluation. In multivariate Cox models, the reversible AO, persistent AO, and PRISm groups were associated with an increased risk of death in crude and adjusted models, except when adjusted for pre-BD FEV_1_ (Table 3). No association with survival was observed in the group of patients with post-BD AO but no pre-BD AO. In the multinomial logistic regression model, the persistent AO and PRISm groups were associated with a greater number of exacerbations when compared with participants with normal spirometry. No association with exacerbations was observed in the post-BD but no pre-BD AO and reversible AO groups. 

**Table 3:** Adjusted association of different lung function groups at baseline with survival, lung function decline, and two or more exacerbations in the previous year in the follow-up evaluation.

	Unadjusted			Adjusted 1^a^		Adjusted 2^b^	
Lung function groups	Risk of Death					
	HR (95% CI)	p	HR (95% CI)	p	HR (95% CI)	p
Normal spirometry (reference)						
Reversible AO (pre-BD reference values)	1.90 (1.1-3.1)	0.01	1.92 (1.2-3.2)	0.01	1.65 (0.9-2.7)	0.056
Persistent AO (pre-BD reference values)	2.99 (2.1-4.3)	< 0.0001	1.81 (1.2-2.6)	0.002	1.21 (0.8-1.9)	0.40
Post-BD AO but no pre-BD AO (pre-BD reference values)	1.04 (0.3-3.3)	0.94	0.75 (0.2-2.4)	0.63	0.61 (0.2-1.9)	0.39
Post-BD AO (post-BD reference values)	1.93 (1.1-3.4)	0.02	1.94 (1.1-3.5)	0.02	1.66 (0.9-2.9)	0.08
PRISm	1.63 (0.9-2.7)	0.06	1.94 (1.2-3.3)	0.01	1.33 (0.8-2.3)	0.32
	Excessive Lung Function Decline					
	b **(95% CI)**	p	b **(95% CI)**	p	b **(95% CI)**	p
Normal spirometry (reference)						
Reversible AO (pre-BD reference values)	−3.07 (−11.7 to 5.5)	0.48	−4.16 (−12.8 to 4.5)	0.35	−7.97 (−16.6 to 0.6)	0.07
Persistent AO (pre-BD reference values)	−5.57 (−12.6 to 1.4)	0.12	−2.20 (−9.3 to 4.9)	0.54	−13.4 (−21.1 to −5.6)	0.001
Post-BD AO but no pre-BD AO (pre-BD reference values)	20.2 (4.9 to 35.5)	0.01	16.3 (0.85 to 31.8)	0.03	10.1 (−5.3 to 25.5)	0.19
Post-BD AO (post-BD reference values)	1.13 (−8.7 to 10.9)	0.82	3.2 (−6.6 to 12.9)	0.51	−1.39 (−11.2 to 8.4)	0.77
PRISm	15 (6.9 to 23.5)	< 0.0001	15 (6.9 to 23.5)	< 0.0001	4.6 (−4.2 to 13.3)	0.30
	Two or more exacerbations in the previous year					
	OR (95% CI)	p	OR (95% CI)	p	OR (95% CI)	p
Normal spirometry (reference)						
Reversible AO (pre-BD reference values)	1.32 (0.4-4.3)	0.65	1.32 (0.4-4.4)	0.66	0.91 (0.3-3.1)	0.88
Persistent AO (pre-BD reference values)	2.39 (1.1-5.2)	0.02	3.57 (1.6-8.2)	0.003	1.09 (0.4-3.1)	0.87
Post-BD AO but no pre-BD AO (pre-BD reference values)	-	-	-	-	-	
Post-BD AO (post-BD reference values)	2.39 (0.8-6.9)	0.11	3.04 (0.9-9.3)	0.051	2.13 (0.7-6.6)	0.19
PRISm	2.88 (1.3-6.6)	0.01	3.12 (1.3-7.3)	0.009	1.11 (0.4-2.9)	0.83

HR: hazard ratio; AO: airflow obstruction; BD: bronchodilator; and PRISm: preserved ratio impaired spirometry. Note 1: The reference group included participants with normal spirometry before and after bronchodilator administration. Note 2: Impact on survival was evaluated with a Cox proportional hazards model. Impact on post-BD excessive FEV_1_ decline (mL/year) was evaluated with multiple linear regression models (with declines being represented by values with a minus sign). Impact on two or more exacerbations in the previous year was evaluated with a logistic regression model. ^a^Age, sex, BMI, comorbidities, number of cigarette packs/day, and number of years of schooling. ^b^Age, sex, BMI, comorbidities, number of cigarette packs/day, number of years of schooling, and FEV_1_.

The persistent AO group was associated with a faster decline in post-BD FEV_1_ in the follow-up evaluation (Table 3), although only in models adjusted by pre-BD FEV_1_ at baseline. The group of patients with post-BD AO, identified only with post-BD reference values, also had a higher adjusted risk of death (hazard ratio = 1.9; 95% CI, 1.1-3.5), but not an accelerated FEV_1_ decline or more frequent exacerbations. 

### Development of incident COPD at follow-up

Individuals in the reversible AO group were at a higher risk of developing a persistent AO pattern (incident COPD) in logistic regression models (OR = 5.7; 95% CI, 3.2-10.2) adjusted for a self-report of asthma (OR = 1.6; 95% CI, 0.9-2.9) and FEV_1_ response to a BD (OR = 2.0; 95% CI, 0.9-4.4). 

## DISCUSSION

The most important findings of the present study, which involved the PLATINO population-based cohort, were as follows. First, a group of 105 individuals (3.6% of the total population) had post-BD AO only if compared with reference values derived from post-BD tests, which represents an additional 46% of individuals identified as having AO with respect to those identified by the pre-BD reference values. Second, in this cohort, in comparison with individuals with normal spirometry, those who met current spirometric criteria for COPD (persistent AO), those with AO only before BD administration (reversible AO), those with a PRISm spirometric pattern, or those with AO identified only with post-BD reference values had an increased risk of death during follow-up. Furthermore, individuals with persistent AO or a PRISm pattern had a higher risk of exacerbations during follow-up, and only those with persistent AO were found with an additional significant FEV_1_ decline (−13.4 mL/year; 95% CI, −5.6 to −21). Third, individuals with reversible AO were at an increased risk of developing COPD during follow-up (OR = 5.7; 95% CI, 3.2-10.2). 

Post-BD spirometry is the current standard for COPD diagnosis; however, spirometry interpretation most commonly is done by comparing against pre-BD reference values, including the widely recommended reference values from the Global Lung Function Initiative^5^ and those derived from the Third U.S. National Health and Nutrition Examination Survey.^7^ Underdiagnosis of post-BD spirometric abnormalities as a result of a comparison with pre-BD reference values has been previously described.^8^ ^,^ ^12^ If AO is defined by a post-BD FEV_1_/FVC ratio below the 5th percentile of reference values, recommended for interpretation of spirometry by American Thoracic Society/European Respiratory Society standards,^3^ ^,^ ^20^ a comparison with post-BD reference values becomes more appropriate: a considerable 28% of underdiagnosis of AO is present if we utilize pre-BD reference values only, which is the usual international practice. 

Malinovschi et al.^10^ assessed the impact of using different reference equations (pre- or post-BD) to define the LLN when interpreting post-BD spirometry values in a general population. The results indicated that approximately twice the number of individuals were classified as having abnormal post-BD spirometry if post-BD reference values were used instead of pre-BD reference values. In addition, individuals who would not have been classified as having impaired FEV_1_ or FEV_1_/FVC because of the reference values demonstrated a higher respiratory burden, more diagnosed COPD, emphysema, and impaired DL_CO_. Our results are in line with the aforementioned study and indicate that the use of post-BD reference values when interpreting post-BD spirometry allows us to identify individuals with AO with frequent exposure to smoking and other respiratory risk factors and decreased survival, that is, with a clinically relevant disease. 

A report of the **S**ub**P**opulations and **I**nte**R**mediate **O**utcome **M**easures **i**n **C**OPD **S**tudy cohort data^14^ investigated whether individuals with variable AO, that is, with AO on pre-BD testing that “reverses” following BD administration, had features of COPD as evaluated by chest CT and whether they progressed to fixed AO over time. The results indicate that participants with reversible AO had 0.3% more emphysema, higher annual FEV_1_ decline, and 6.2 times the hazard of developing COPD than those without AO at baseline. 

In the present study, a total of 427 individuals (14.6%) had pre-BD AO, 273 (9.3%) had post-BD AO (vs. pre-BD reference values), and 378 (12.9%) had AO when compared with post-BD reference values, with some showing no AO on the pre-BD test. The three groups were associated with increased risk of death and risk of exacerbations but showed normal FEV_1_ decline in a crude and adjusted analysis; after taking into account FEV_1_, we found that only the persistent AO and reversible AO (borderline) groups were still associated with an increased risk of death. Furthermore, persistent AO was associated with an accelerated FEV_1_ decline. The group of patients with post-BD AO but no pre-BD AO was small (n = 43) and their FVC increased significantly after BD administration; that is, they were more often volume responders, they had a longer forced expiratory time after BD administration than before BD administration, and they showed more heterogeneity in test quality, conditions that added up to fulfill the criteria for post-BD AO. A bronchoconstriction agent response to BD administration is unlikely, given that their mean FEV_1_ also increased after BD administration. 

Importantly, the post-BD spirometric results in our study decreased intra-test variability when compared with pre-BD testing, supporting the higher reliability of post-BD tests. In addition, individuals in the group had a higher prevalence of symptoms, a prior diagnosis of tuberculosis, asthma, COPD, and comorbidities, suggesting a greater specificity for disease than pre-BD AO. For the same reason, it is considered that the decline of post-BD FEV_1_ is relatively more consistent than the pre-BD test.^21^ ^,^ ^22^

Individuals with pre-BD AO are at a higher risk of death and should be followed over time, given that they are also at a greater risk of fulfilling the criteria for a COPD diagnosis in the future. However, the prevalence of pre-BD AO is approximately 30% higher than the current post-BD criteria, and this could generate logistical difficulties in addressing an increase in COPD patients generated only by a tentative change in the definition. A better alternative is to consider reversible AO as a group of individuals at risk of developing COPD (i.e., pre-COPD patients) and intervene with smoking cessation, control of other risk factors, and close monitoring. 

Some of the limitations that must be taken into account when interpreting our results are as follows. The study accomplished only one follow-up evaluation in three of the five cities reported in the baseline study, although most of the baseline participants in those three cities were evaluated, and the final sample is still considerable. The total number of patients analyzed was further reduced because we had to include only those with post-BD tests in the first and second evaluations. Because this was a population-based study, participants with severe spirometric abnormalities were scarce in comparison with cohorts of patients or tobacco smokers; however, our results reflect more closely the conditions of the entire population, and we had a large control group comprising individuals with normal spirometry, never smokers, and individuals lacking other relevant exposures often absent or scarce in patient cohorts. Individuals with pre-BD AO as defined by the LLN have an increased risk of death and developing COPD (defined as post-BD AO), and they should be followed over time and recommended preventative measures. Using post-BD reference values to classify individuals evaluated after BD administration identifies a group of individuals who have relevant exposures and who are at an increased risk of exacerbations and death. 
